# Reply to: Stroke awareness in a Brazilian Northeastern capital city and the burden of the COVID-19 pandemic

**DOI:** 10.1055/s-0045-1806920

**Published:** 2025-04-22

**Authors:** Letícia Januzi de Almeida Rocha, Monica Thalia de Brito Melo, Renata Girardi Piva, Samira Mercaldi Rafani, Octavio Marques Pontes-Neto, Eva Rocha, Jussara Almeida de Oliveira Baggio

**Affiliations:** 1 Universidade Federal de Alagoas, EBSERH, Faculdade de Medicina, Hospital Universitário Professor Alberto Antunes, Maceió AL, Brazil.; 2 Universidade Federal de Alagoas, Curso de Medicina, Arapiraca AL, Brazil.; 3 Universidade de São Paulo, Faculdade de Medicina de Ribeirão Preto, Departamento de Neurociências e Ciências do Comportamento, Ribeirão Preto SP, Brazil.; 4 Universidade Federal de São Paulo, Escola Paulista de Medicina, Departamento de Neurologia, São Paulo SP, Brazil.

Dear Editor,

We have received with interest the comments made by the authors DGSM, and TGSM. regarding our article “Stroke awareness in a Brazilian Northeastern capital city and the burden of the COVID-19 pandemic.” 1 2

Indeed, the use of artificial intelligence for the early detection of retinal changes and its strong correlation with classical risk factors for cerebrovascular diseases appears to be a promising strategy that may soon be utilized in risk screening for conditions such as stroke and acute myocardial infarction. Although retinal changes can also be observed in individuals with impaired glucose tolerance and recent-onset diabetes, 3 they are more prevalent in chronic stages of diseases such as diabetes mellitus and systemic arterial hypertension. These conditions are often associated with each other and with other comorbidities in the same patient.

Gong et al. 4 published a meta-analysis in 2023 that estimated the relative risk (RR) of stroke in patients with diabetic retinopathy without other classical risk factors. The RR was 1.70 (95% confidence interval [CI]: 1.43–2.03), which was lower than that observed in those with additional associated conditions (2.29; 95% CI: 0.93–5.65). Thus, we believe that this tool could serve as an additional method for assessing the severity of these diseases and, consequently, assisting in strategies for preventing secondary conditions (stroke and myocardial infarction) and complications (severe retinopathy with a risk of vision loss).

Another aspect we would like to highlight is the effectiveness of this tool as a population-wide strategy. Unfortunately, access to preventive healthcare strategies remains limited in Brazil. Studies indicate that less than 60% of diabetic patients, for example, undergo routine ophthalmologic examinations at the intervals recommended by current guidelines. 5 The reasons for this include both the countrywide lack of access to specialized healthcare services and the absence of effective strategies for risk factor management and lifestyle changes. Thus, despite its promising nature due to practicality and the lack of need for a specialist, we believe that cost-effectiveness studies need to be conducted.

The World Stroke Organization recommends the implementation of both individual and population-level strategies for primary stroke prevention. The use of technology is a potential approach to improve the identification and management of individuals at high risk for cardiovascular diseases. 6 However, studies on feasible and effective strategies within the Brazilian context are necessary, taking into account the country's socioeconomic diversity.

In conclusion, we consider early and periodic screening for retinal changes to be of utmost importance, given its strong correlation with severe, costly, and disabling cerebrovascular outcomes. However, we emphasize that early monitoring of classical risk factors through well-established, simple measures—such as blood pressure monitoring, frequent glucose level checks, and combating obesity—remains the foundation of prevention strategies, as these factors typically precede the onset of retinopathy.
